# 3D Cell Culture-Based Global miRNA Expression Analysis Reveals miR-142-5p as a Theranostic Biomarker of Rectal Cancer Following Neoadjuvant Long-Course Treatment

**DOI:** 10.3390/biom10040613

**Published:** 2020-04-16

**Authors:** Linas Kunigenas, Vaidotas Stankevicius, Audrius Dulskas, Elzbieta Budginaite, Gediminas Alzbutas, Eugenijus Stratilatovas, Nils Cordes, Kestutis Suziedelis

**Affiliations:** 1 National Cancer Institute, LT-08660 Vilnius, Lithuania; linaskunigenas@gmail.com (L.K.); audrius.dulskas@nvi.lt (A.D.); elzebudg@gmail.com (E.B.); eugenijus.stratilatovas@nvi.lt (E.S.); 2 Life Sciences Center, Institute of Biosciences, Vilnius University, LT-08412 Vilnius, Lithuania; 3 Life Sciences Center, Institute of Biotechnology, Vilnius University, LT-08412 Vilnius, Lithuania; 4 Institute of Clinical Medicine, Faculty of Medicine, Vilnius University, LT-08406 Vilnius, Lithuania; 5 University of Applied Sciences, Faculty of Health Care, LT-08303 Vilnius, Lithuania; 6 Thermo Fisher Scientific, LT-02241 Vilnius, Lithuania; gediminas.alzbutas@thermofisher.com; 7 Institute of Informatics, Faculty of Mathematics and Informatics, Vilnius University, LT-08303 Vilnius, Lithuania; 9 Department of Radiation Oncology, University Hospital Carl Gustav Carus, Technische Universität, Dresden, Germany; 8 OncoRay—National Center for Radiation Research in Oncology, Faculty of Medicine, Technische Universität, D–01307 Dresden, Germany; Nils.Cordes@OncoRay.de; 10 Helmholtz–Zentrum Dresden–Rossendorf, Institute of Radiooncology–OncoRay, D–01328 Dresden, Germany; 11 German Cancer Consortium (DKTK), partner site Dresden, D–69192 Heidelberg, Germany; 12 German Cancer Research Center (DKFZ), D–69192 Heidelberg, Germany

**Keywords:** colorectal carcinoma, rectal cancer, 3D cell culture, miRNA, tumor microenvironment, cell adhesion, cancer biomarkers, neoadjuvant therapy, miR-142

## Abstract

Altered expression of miRNAs in tumor tissue encourages the translation of this specific molecular pattern into clinical practice. However, the establishment of a selective biomarker signature for many tumor types remains an inextricable challenge. For this purpose, a preclinical experimental design, which could maintain a fast and sensitive discovery of potential biomarkers, is in demand. The present study suggests that the approach of 3D cell cultures as a preclinical cancer model that is characterized to mimic a natural tumor environment maintained in solid tumors could successfully be employed for the biomarker discovery and validation. Subsequently, in this study, we investigated an environment-dependent miRNA expression changes in colorectal adenocarcinoma DLD1 and HT29 cell lines using next-generation sequencing (NGS) technology. We detected a subset of 16 miRNAs differentially expressed in both cell lines cultivated in multicellular spheroids compared to expression levels in cells grown in 2D. Furthermore, results of in silico miRNA target analysis showed that miRNAs, which were differentially expressed in both cell lines grown in MCS, are involved in the regulation of molecular mechanisms implicated in cell adhesion, cell-ECM interaction, and gap junction pathways. In addition, integrins and platelet-derived growth factor receptors were determined to be the most significant target genes of deregulated miRNAs, which was concordant with the environment-dependent gene expression changes validated by RT-qPCR. Our results revealed that 3D microenvironment-dependent deregulation of miRNA expression in CRC cells potentially triggers essential molecular mechanisms predominantly including the regulation of cell adhesion, cell–cell, and cell–ECM interactions important in CRC initiation and development. Finally, we demonstrated increased levels of selected miR-142-5p in rectum tumor tissue samples after neoadjuvant long course treatment compared to miR-142-5p expression levels in tumor biopsy samples collected before the therapy. Remarkably, the elevation of miR-142-5p expression remained in tumor samples compared to adjacent normal rectum tissue as well. Therefore, the current study provides valuable insights into the molecular miRNA machinery of CRC and proposes a potential miRNA signature for the assessment of CRC in further clinical research.

## 1. Introduction

Colorectal cancer (CRC) is the most common malignancy of the gastrointestinal tract. Approximately 1.23 million new cases are estimated each year worldwide, which defines CRC as the third leading oncologic disease after lung and breast cancer [1]. The delayed diagnosis of CRC is the major impediment to successful anti-cancer therapy. Early detected CRC is effectively cured by surgical tumor resection, meanwhile, cancer diagnosed at advanced stages leads to poor 12.5% five-year survival, regardless of anti-cancer treatment applied [2]. The progression of colorectal cancer is an asymptomatic process and its clinical manifestations are observed only when the adjacent tissues are invaded. Another challenging feature of CRC is its exclusively rapid development, which exacerbates full recovery even when the early stage is detected. Hence, the early diagnosis, prognosis, and evaluation of the current status of disease development of CRC could be greatly facilitated by cancer-specific molecular biomarkers [3].

A current clinical diagnosis of CRC is based on the assessment of several cancer-specific molecular biomarkers including carcinoembryonic antigen (CAE) and CA19-9. However, these molecules do not provide reliable diagnostic results due to insufficient specificity and sensitivity for the tumor tissue [4]. Therefore, the identification of novel highly sensitive molecular biomarkers remains a quintessential aim. A recent discovery of cancer-related and tissue-specific deregulation of miRNAs has suggested its further potential applicability in cancer detection and treatment [5]. Furthermore, a small size, inherent stability, and the resistance to RNAse hydrolysis represent the miRNAs as appropriate molecular markers. In addition, their extreme stability and predictive properties in biological fluid samples further encourage the translation of miRNAs as potential biomarkers in the clinical practice [6].

The research of potential cancer biomarkers raises the demand for a proper experimental design. However, the discovery of novel biomarkers requires hundreds of high-quality patient samples reducing the ability to screen potential biomarkers in large-scale [7]. Besides, validation of reproducible, specific and sensitive biomarkers is limited by case-specific disease progression in patients usually undergoing long term therapy and various bio-specimen characteristics [8,9]. These restrictions have provoked the approach of in vitro cell line models approximating the characterization of potential tumor markers. However, the most common two-dimensional (2D) cell culture in vitro models typically grown on 2D plastic surfaces which is far different from the native tumor environment resulting in poor clinical relevance. Thus, to achieve clinically relevant results, a three dimensional (3D) tumor environment in vivo remains to be taken into consideration. For this reason, recently developed 3D cell culture models are extensively applied in cancer research for its ability to closely mimic the tumor microenvironment including the reflection of native cellular morphology, proliferation rates and gene expression patterns observed in tumor tissue in vivo [10,11]. Previous reports indicated that the drug response of tumor cells grown in 3D cell cultures is more consistent with tumor resistance compared to cells cultivated in 2D [12,13,14]. Therefore, this suggests that the applications of 3D cell culture models are necessary to discover clinically relevant biomarkers leading to the advancements in the development of anticancer therapies and more personalized medicine.

To address this question, in the present study we applied a 3D multicellular spheroid cell culture model and investigated the microenvironment-dependent changes of miRNA expression in colorectal carcinoma DLD1 and HT29 cell lines. Next-generation sequencing (NGS) technology was employed for the analysis of miRNA expression levels in CRC cells cultivated in 3D multicellular spheroid (MCS) and 2D cell cultures. The analysis revealed 16 miRNAs commonly expressed in both CRC cell lines in a 3D-dependent manner. Furthermore, target analysis of differentially expressed miRNAs suggested the potential implication of certain miRNAs in the regulation of molecular CRC processes including cell adhesion, cell–ECM interactions. A consecutive gene expression analysis of potential miRNA targets elucidated gene expression changes in cells cultivated under 3D cell culture conditions which corresponded to our bioinformatical in silico analysis. To validate our model, we evaluated the expression levels of 5 selected miRNAs, (miR-26b-5p, miR-142-5p, miR-194-5p, miR-301a-5p and miR-3074-5p) in tumor samples (*n* = 72) collected from 24 patients. The analysis revealed increased levels of selected miRNA miR-142-5p in rectum tumor tissue samples after neoadjuvant long course treatment compared to miR-142-5p expression levels in tumor biopsy samples collected before the therapy. The elevation of miR-142-5p expression remained in tumor samples compared to adjacent normal rectum tissue as well. In conclusion, the profile of differentially expressed miRNAs determined in this study could have potential diagnostic and therapeutic applications assessing the patients with CRC.

## 2. Materials and Methods

### 2.1. Cell Lines

Human colorectal carcinoma DLD1 (CCL-221^TM^) and HT29 (HTB-38^TM^) cell lines were obtained from the American Type Culture Collection (Rockville, Maryland, USA). The cells were maintained in RPMI-1640 (DLD1) and DMEM (HT29) cell culture media (ThermoFisher Scientific, Waltham, Massachusetts, USA) respectively, supplemented with 10% fetal bovine serum (ThermoFisher Scientific), 2mM glutamine (ThermoFisher Scientific), 1mM sodium pyruvate (ThermoFisher Scientific ), 100 UI/mL penicillin (Merck, Darmstadt, Germany) and 0.1 mg/mL streptomycin (Merck). CRC cell cultures were maintained at 37 °C in a humidified atmosphere containing 5% CO_2_.

### 2.2. Cell Culture Models

All experiments were performed following 6 days of cell growth and repeated at least three times. Cell culture media were changed every second day. The 2D monolayers were obtained by plating 3.5 × 10^4^ DLD1 and 1.0 × 10^5^ HT29 cells in 25 cm^2^ plastic cell culture flasks. Three-dimensional (3D) multicellular spheroids (MCSs) were formed as described previously [15] with minor modifications. Briefly, 7.0 × 10^3^ DLD1 and 3.5 × 10^3^ HT29 cells were suspended in 200μL cell culture medium then plated in each well of 96 round-bottom well plates and centrifuged at 1000× *g* for 10 min. To prevent cell attachment to the surface of the culture plates, each round-bottomed well was pre-coated with a layer of 1% agarose solution in sterile water. Cells were photographed with an inverted optical microscope Eclipse TS100 (Nikon, Tokyo, Japan) and digital camera DS-Fi2 (Nikon), 2 and 6 days after seeding. The size of multicellular spheroids was assessed by measuring spheroid diameter using SpheroidSizer 1.0 as described previously [16]. Multicellular spheroids that reached 400 ± 20 µm diameter 2 days after cell platting were further cultivated for the experiments.

### 2.3. Patient Samples

The study was approved by the Ethics Committee of Vilnius Region Biomedical Research (2017-07-04; No. of permission 158200-17-930-433) and informed consent was obtained from all participants. All clinical procedures were carried out at the National Cancer Institute in Lithuania between 2017–2019 according to Helsinki regulation. Patients diagnosed with rectal cancer received neoadjuvant long-course chemoradiotherapy which included 25–28 fractions of irradiation (total dose of 45–51 Gy) and fluorouracil based treatment during 5 week period. Tumour and adjacent normal rectum tissue samples were collected during surgical tumor resections 8–12 weeks after the neoadjuvant treatment and stored at −80°C in RNAlater (ThermoFisher Scientific) until needed. The sample cohort contained three groups and included rectal tumor samples collected from patients before long course neoadjuvant treatment (n=24), normal (*n* = 24) and tumor tissue (*n* = 24) samples collected from the same patients after the therapy. Patient demographic and clinical characteristics are summarised in Table 1.

### 2.4. RNA Extraction

Total RNA enriched in small non-coding RNAs was isolated from tumor tissue samples or from 1 × 10^6^ of CRC cells grown in monolayer and MCS 6 days after cell plating using MirVana miRNA isolation Kit (ThermoFisher Scientific) according to manufacturer’s instructions. The quantity and quality of total RNA were evaluated using Nanodrop (ThermoFisher Scientific). Subsequent RNA integrity assessment, based on the evaluation of 28S and 18S ribosomal subunit peaks in electropherogram, was performed using Bioanalyzer 2100 (Agilent, Santa Clara, California, USA). Total RNA samples with A260/280 ratio of more than 1.95 and a high RNA integrity number were used for further experiments.

### 2.5. miRNA Library Sequencing and NGS Data Proceeding

2 μg of total RNA were applied for small RNA sequencing library preparation using the NEXTflex small RNA sequencing kit (Bioo Scientific, Austin, Texas, USA) according to the manufacturer’s instructions. High-throughput sequencing of prepared small RNA libraries was performed using Illumina HiSeq 2500 sequencer (Illumina, San Diego, California, USA) at BaseClear sequencing facility (BaseClear, Leiden, The Netherlands). Reads and its corresponding quality scores provided by the next-generation sequencing were stored in FASTQ files using CASAVA pipeline v.1.8.3. Qualitative evaluation of high throughput sequencing data was performed using FastQC v0.10.1 as described previously [17]. 3‘ adapter sequences and random 4N bases wrapping each read were trimmed using Cutadapt v1.9.1 [18] and reads with length less than 15 nucleotides and the quality score less than 25 were excluded from further analysis. Remaining sequences were aligned to the human genome GRCh37 using short read alignment tool Bowtie2v.2.2.9 [19] and mature miRNAs were identified by aligning the mapped reads to the annotated miRNA sequences from miRBase v21 data depository [20].

To evaluate miRNA expression changes in cells cultivated in 2D and 3D environments, reads of each miRNA were analyzed using Bioconductor’s edgeR software package v.3.4.2 [21]. Read counts were normalized using the upper quartile method and p values were adjusted with the Benjamini-Hochberg method. Statistical analysis was conducted using Fisher exact test. MiRNAs with at least 1.5 fold change, not less than 10 reads per million transcripts in miRNA library and *p*-value < 0.05 were considered significantly differentially expressed. High-throughput sequencing data are available at the GEO database (Accession No. GSE112492).

### 2.6. Functional miRNA Target Analysis

The potential regulatory mechanisms of differentially expressed miRNAs in CRC cells cultivated in 3D MCSs were assessed by using in silico bioinformatical tools. Firstly, to provide a set of the most relevant candidate gene targets of the corresponding miRNAs, two strategies were employed to collect two distinct groups of experimentally validated and in silico predicted miRNA targets. This separated data analysis design was preferred because the set of evidently validated miRNA target genes is limited on experimental data collected in database. Meanwhile, target gene prediction tools could generate thousands of statistical miRNA-mRNA interactions lacking biological evidence. Strong experimental evidence-based miRNA targets were identified using miRTarBase database [22]. Meanwhile, predicted miRNA targets were analyzed using DIANA Tools web server and microT-CDS v5.0 toolkit (http://diana.imis.athena-innovation.gr/) [23,24]. miRNA targeted genes score (miTG score) of 0.8 was determined as a minimum threshold value for microT-CDS algorithm. Secondly, to extract key pathways associated with miRNAs differentially expressed in CRC cells, KEGG pathway enrichment analysis was performed using obtained target gene sets. KEGG categories enriched in strong experimental evidence-based miRNA targets were assessed using WebGestalt 2017 online resource [25,26], meanwhile DIANA mirPath v.3 online enrichment tool [27] was employed for the enrichment analysis of predicted miRNA targets. *p* values were calculated using the hypergeometric test and adjusted with multiple Benjamini and Hochberg testing. Functional categories were considered as significantly enriched if at least 5 genes were assigned and the corrected *p*-value was lower than 0.05. Thirdly, to identify differentially expressed leading strand miRNAs located in polycistronic miRNA clusters, data provided in miRBase [20] was analyzed. Differentially expressed miRNAs encoded in less than 10kb distance and located in the same DNA strand were considered to be encoded in a polycistronic miRNA cluster.

### 2.7. Differential miRNA Expression Analysis by RT-qPCR

For miRNA sequencing data validation, the expression of mature miRNAs was quantified by RT-qPCR method. cDNA synthesis was performed using Revert Aid RT Kit (ThermoFisher Scientific) according to manufacturer‘s instructions with minor changes as described previously [28]. Briefly, each reaction mixture included 4 µL of reaction buffer, 0.8 µL of dNTP mix, 0.8 µL of 0.25 µM stem-loop RT primer, 0.4 µL of Ribolock RNase inhibitor, 1 µL of 200 ng/µL RNA, 12 µL of nuclease-free water and 200U of Revert Aid Reverse Transcriptase. RT-qPCR was performed using Realplex4 Mastercycler thermocycler (Eppendorf, Hamburg, Germany) and Luminaris HiGreen qPCR Master Mix (ThermoFisher Scientific) according to the manufacturer’s instructions. Briefly, for each reaction in a 96 well plate, 1 μL of 4 times diluted cDNA, 5μL Luminaris HiGreen qPCR Master Mix, 3.4 μL nuclease-free water and 0.3 μM forward and reverse primer was used. Reaction cycling conditions included polymerase activation at 95 °C for 3 min, followed by 3 cycles of denaturation at 95 °C for 15 s, annealing at 55 °C for 1 min and extension at 60·°C for 30 s. These steps were then followed by 40 cycles of 2-step amplification consisting of denaturation at 95 °C for 15 s, annealing, and extension at 60 °C for 15 s. The relative changes of miRNA expression were calculated by ΔΔCt method and U6 and RNU48 snRNAs were used as an internal control to normalize the miRNA expression. Primer sequences used for gene expression analysis are shown in Additional file 1 Table S1 and S2.

### 2.8. Differential Predicted Target Gene Expression Analysis by RT-qPCR

To determine gene expression changes of potential miRNA targets in cells cultivated in a 3D environment, RT-qPCR was performed. cDNA was synthesized using 1μg of total RNA and Revert Aid RT Kit (ThermoFisher Scientific) according to manufacturer‘s instructions. Random hexamer primers included in the manufacturer‘s kit were used following the protocol. RT-qPCR was performed using Realplex4 Mastercycler thermocycler (Eppendorf) and KAPA SYBR FAST qPCR Kit (KAPA Biosystems, Wilmington, Massachusetts, USA) according to the manufacturer’s instructions. Briefly, for each reaction in a 96 well plate, 1 μL of 10 times diluted cDNA, 5μL KAPA SYBR FAST Master Mix, 3.8 μL nuclease-free water and 0.2 μL of 10 μM forward and reverse primer mix was used. Reaction cycling conditions included polymerase activation at 95 °C for 3 min, followed by 40 2-step amplification cycles consisting of denaturation at 95 °C for 15 s and annealing/extension at 60 °C for 30 s. The relative changes of gene expression were calculated by ΔΔCt method and TBP housekeeping gene was used as an internal control to normalize gene expression. Primer sequences used for gene expression analysis are shown in Additional file 1 Table S3.

### 2.9. Statistical Analysis

The statistical significance of gene expression differences in cells cultivated in 3D versus 2D cultures was calculated using an unpaired *t*-test. Differences in miRNA expression levels in tumor tissue samples were evaluated using paired *t*-test. The observations with a *p*-value of less than 0.05 were considered significant. Each observation is represented as the mean ± standard deviation of three independent biological experiments.

## 3. Results

### 3.1. The Three-Dimensional Environment Promotes Global miRNA Expression Changes in CRC Cell Lines

**Figure 1:**
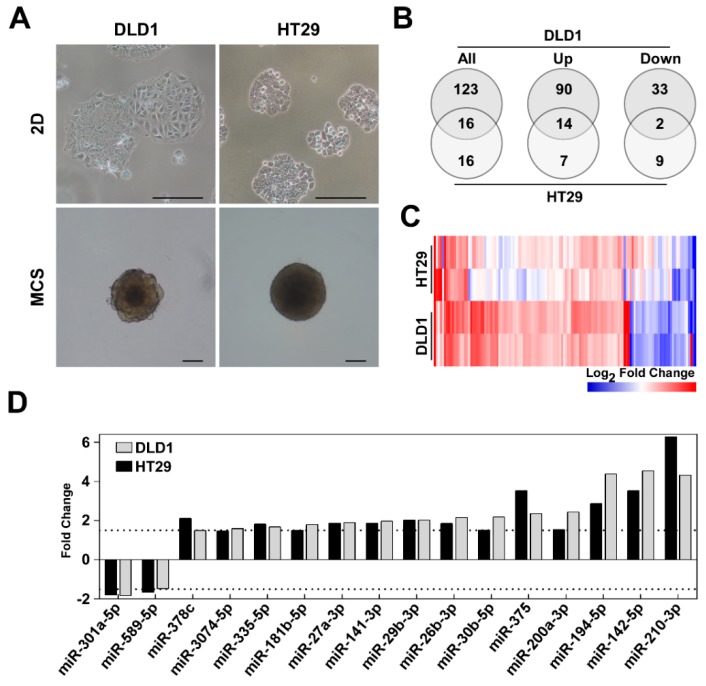
Different patterns of cell morphology and miRNA expression in colorectal adenocarcinoma DLD1 and HT29 cell lines cultivated in 3D multicellular spheroids (MCS) and 2D monolayer. (**A**) Phase-contrast photomicrographs of DLD1 and HT29 cells cultivated in 2D monolayer (upper panel) and MCS (lower panel). Cells were cultivated for 6 days before imaging, scale bar-250μm. (**B**) Venn diagrams representing statistically significant changes of miRNA expression (>1.5 fold change, *p* < 0.05, at least 10 mapped reads per million transcripts in miRNA library) in DLD1 and HT29 cells cultivated in MCS. (**C**) Heatmap demonstrating different miRNA expression patterns in DLD1 and HT29 cell lines (>1.5 fold change, FDR < 0.05). (**D**) Differentially expressed miRNAs in both cell lines cultivated in MCS. 2D—monolayer cell culture; MCS—multicellular spheroid culture.

The cell growth in MCS is followed by the formation of the heterogeneous cellular zones within spheroid which was highly anticipated in the present study. Thus, to observe fully developed characteristic proliferation zones in MCS, CRC cells were cultivated for 6 days. On day 6, the cells (Figure 1A) exhibited a distinctive flat morphology and formed flat colonies which reached 80% confluence under 2D conditions. Meanwhile, in the MCS culture cells formed 3D non-adherent spheroids which reached the diameters of 548 ± 10 μm for DLD1 and 582 ± 10 μm for HT29 cells, respectively. Then, to identify the microenvironment-dependent miRNA expression changes in CRC cells grown in MCS and 2D, Illumina’s high-throughput sequencing platform for miRNA expression profiling was applied.

The NGS obtained a total read count of 11 to 36 million reads per sample for DLD1 and HT29 cells grown in MCS and 2D (Additional file 2 Table S1). A total of 90–98% of raw reads passed quality and length control following adapter removal for DLD1 and HT29 miRNA library samples. Approximately 98% of these reads were mapped to the human genome. The length distribution indicated that the majority of annotated reads of DLD1 cell samples were 22 nucleotides in length (Additional file 2 Figure S1A). Mapping the reads to the mature miRNAs showed that the read-length peak of 23 nucleotides observed in HT29 samples was due to a high content of mature 23 nt length miRNA sequences including miR-10a-5p, miR-200b-5p, and miR-20a-5p. In addition, the distribution of annotated reads showed that approximately 87% of mapped reads were attributed to mature miRNA sequences in DLD1 cell samples and over 90%—in HT29, showing a significant library enrichment in mature miRNAs (Additional file 2 Figure S1B). Next, the differences of miRNA transcript levels in CRC cells cultivated in 3D and 2D cultures were evaluated using an edgeR-based R pipeline defining those miRNAs as significantly differentially expressed with at least 1.5 fold change, not less than 10 transcripts per million reads in miRNA library and *p*-value < 0.05. Based on these statistical significance parameters, the NGS analysis revealed a total of 155 miRNAs were differentially expressed in CRC cells grown in MCS compared to the expression levels in cells cultivated in 2D (Figure S1B, Additional file 3).

Among these, a total of 139 differentially expressed miRNAs were identified in DLD1 and 32 miRNAs—in HT29. Approximately 75% and 66% of differentially expressed miRNAs were up-regulated in DLD1 and HT29 cells grown in MCS, respectively. To elucidate the distribution of miRNA expression profiles in DLD1 and HT29 cells cultivated in MCS, hierarchical clustering analysis was performed. The heatmap showed more robust changes in miRNA expression in DLD1, than in HT29 cells cultivated in MCS (Figure 1C). Furthermore, a total of 16 differentially expressed miRNAs which were expressed in both cell lines in an environment-dependent manner is depicted in Figure 1D. To determine the most distinctive miRNA expression discrepancies, we enlisted the top10 differentially expressed (up- or down-regulated) miRNAs in DLD1 and HT29 cells cultivated in MCS (Additional file 2 Table S2). DLD1 cells cultured in MCS exhibited a distinct up-regulation of miR-1246 and miR-9 and a robust down-regulation of miR-371b-3p. Meanwhile, in HT29 cells, miR-210-3p was the most up-regulated and miR-934—down-regulated miRNAs.

### 3.2. Differentially Expressed miRNAs Are Potential Molecular Modulators of Cell Adhesion

**Figure 2:**
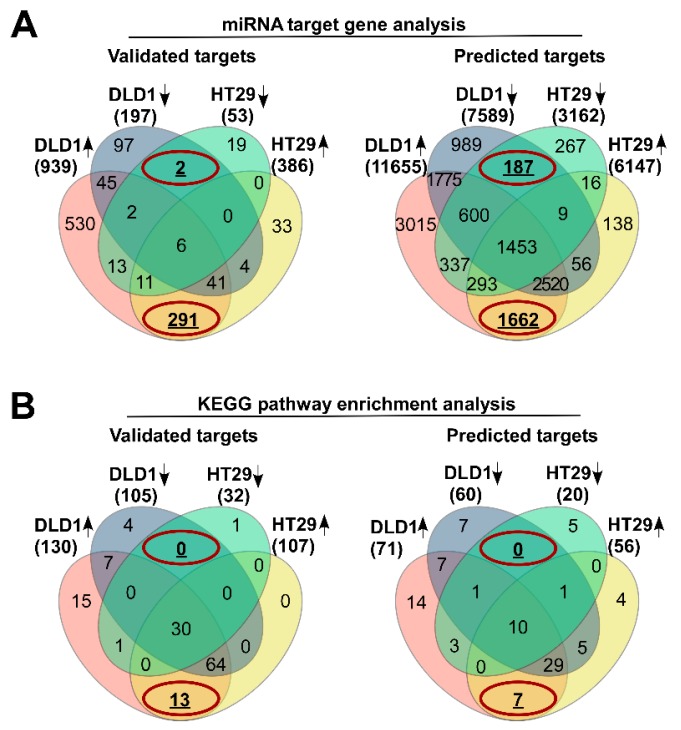
In silico analysis of differentially expressed miRNAs. (**A**) Venn diagrams representing the experimentally validated (left) and predicted (right) miRNA target genes. Superimposable unique genes targeted by differentially expressed miRNAs in DLD1 and HT29 cells are highlighted in circles. (**B**) Results of KEGG pathway enrichment analysis. Pathways enriched in experimentally validated miRNA target genes (left) and putative targets (right) are represented in the form of Venn diagrams and overlapping results in DLD1 and HT29 cell lines are indicated in red circles.

For a better comprehension of biological behavior of differentially expressed miRNAs, in silico analysis of strong experimental evidence-based and putative target genes was performed using miRTarBase and microT-CDS databases, respectively. The results of miRNA target gene analysis demonstrated that up-regulated miRNAs are potentially implicated in the regulation of a considerably higher amount of genes than down-regulated miRNAs (Figure 2A). These results are concomitant with an observation that target genes common for both cell lines are predominantly associated with up-regulated miRNAs. According to Venn diagram analysis, a total of 291 experimentally validated (Figure 2A, left diagram) and 1662 predicted targets (Figure 2A, right diagram) of up-regulated miRNAs were common for both cell lines. Whereas only 2 validated and 187 predicted target genes were identified as presumably targeted by down-regulated miRNAs.

Next, KEGG pathway enrichment analysis was performed by integrating experimentally validated and predicted miRNA target genes using WebGestalt and mirPath online tools. Enrichment significance was considered if the false discovery rate (FDR) was estimated lower than 0.05 and the functional group was enriched in at least 5 target genes. The results of functional group enrichment represented in Venn diagrams (Figure 2B) have elucidated KEGG pathways, distinctively enriched in presumable target genes of differentially up-regulated miRNAs: 13 unique functional groups were enriched in experimentally validated miRNA target genes (Figure 2B, left diagram) and 7 molecular mechanisms were enriched in predicted target genes (Figure 2B, right diagram). Among these, ECM-receptor interaction and Gap junction were enriched both in experimentally validated and predicted target genes of differentially up-regulated miRNAs in CRC cells grown in MCS (Additional file 4 Table S1). For example, in silico pathway enrichment analysis results (Additional file 4 Table S2) indicated that miR-29a-3p, miR-29b-3p, and miR-29c-3p differently expressed in DLD1 cells are strongly implicated in the regulation of ECM-receptor interaction. The deregulated expression of miR-29b-3p, miR-194-5p, miR-210-3p and miR-335-5p could be associated with this molecular mechanism in both cell lines. In addition, miRNA target analysis insinuated a potential regulation of integrin ITGA6, ITGA9, ITGB1 and collagen COL4A2, COL1A1, COL4A1 gene expression, meanwhile target genes associated with gap junction category included platelet-derived growth factors and its receptors (Additional file 4 Table S2).

### 3.3. Aberrantly Expressed miRNAs in Both DLD1 and HT29 Cell Lines Are Associated With ECM and Gap Junction Signaling Maintenance

A further analysis was oriented towards the miRNAs which were significantly differentially expressed in both colorectal adenocarcinoma DLD1 and HT29 cell lines cultivated in MCS culture compared to 2D. In this study, a total of 16 miRNAs were detected, which were differentially expressed in both cell lines. Among these, 14 miRNAs were up-regulated and 2 miRNAs—down-regulated in CRC cells. For a detailed examination of the potential regulatory roles of these miRNAs, KEGG pathway enrichment analysis was employed for miRNA target prediction. The results indicated a significant enrichment in functional categories of cell adhesion, including ECM-receptor interaction, focal adhesion, regulation of actin cytoskeleton and formation of gap junctions (Additional file 5 Table S1). Furthermore, a robust enrichment was observed in molecular pathways that regulate the development of colorectal adenocarcinoma including PI3K-Akt, Ras, TGF-beta and Wnt signaling pathways. Consequently, we sought to identify which miRNAs are the most feasible regulators of these functional groups. Further target analysis of validated miRNA-target gene interactions found that 6 miRNAs—miR-200a-3p, miR-141-3p, miR-375, miR-194-5p, miR-27a-3p, and miR-30b-5p—are potentially involved in the regulation of colorectal carcinoma development, whereas miR-29b-3p and miR-335-5p were presumably associated with regulation of cellular adhesion processes (Additional file 5 Table S2).

**Figure 3:**
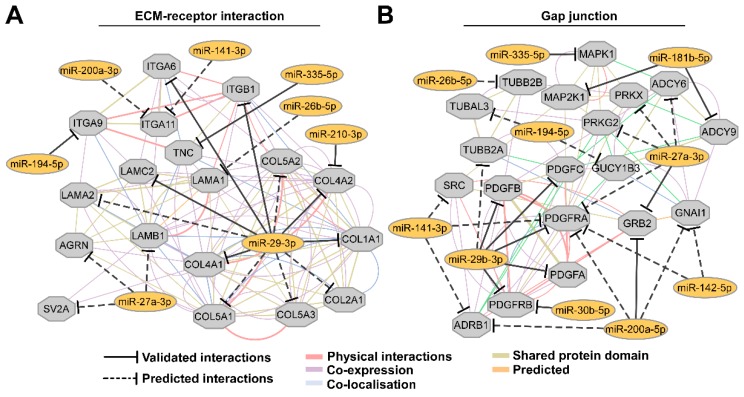
A network of putative miRNA-mRNA interactions involved in cell adhesion. (**A**) Differentially expressed miRNAs in both cell lines which are involved in ECM-receptor interaction. (**B**) Network showing the miRNA involvement in the regulation of gap junctions. PPI networks were generated using GeneMANIA plugin for Cytoscape platform. Only the unique potential target genes of up-regulated miRNAs in both cell lines are represented.

Next, the predicted miRNA-mRNA interaction networks, involved in the regulation of cell adhesion, were visualized using GeneMANIA plugin for Cytoscape platform. The visualization of the ECM-receptor interaction regulatory network elucidated that besides miR-29b-3p and miR-335-5p, miR-27a-3p is also potentially significantly involved in this molecular process (Figure 3A). In addition, the network of gap junction regulatory process has demonstrated a considerable predicted participation of miR-181-5p, miR-141-3p, miR-200a-3p, miR-194-5p and miR-142-5p (Figure 3B).

### 3.4. miRNAs of the Polycistronic miR-23a/27a/24-2 Cluster are Up-Regulated in CRC Cells in a 3D-Specific Manner

Further, we aimed to investigate whether at least two differentially expressed miRNAs observed in our study are located in clustered regions. The association of miRNA into polycistronic clusters was determined according to data provided in miRBase regarding the localization of other adjacent miRNAs in the same DNA strand in less than 10kb distance. The database analysis revealed that miRNAs differentially expressed in DLD1 cells were located in 18 polycistronic clusters (Table 2, Additional file 6 Table S1). Meanwhile, only 2 polycistronic clusters of differentially expressed miRNAs were detected in HT29 cells. Interestingly, at least two miRNAs encoded in the miR-23a/27a/24-2 cluster of 19 chromosome were differentially expressed in both cell lines cultivated in 3D multicellular spheroids (Additional file 6 Table S2). KEGG pathway enrichment analysis was performed to examine potential biological mechanisms regulated by the leading strand miRNAs located in the miR-23a/27a/24-2 cluster. The results demonstrated that functional gene groups implicated in cell adhesion including Focal adhesion and Proteoglycans in cancer were enriched in experimentally validated and predicted target genes of all miRNAs located in this cluster (Additional file 6 Table S3).

### 3.5. Validation of NGS and miRNA Target Gene Data Analysis

**Figure 4:**
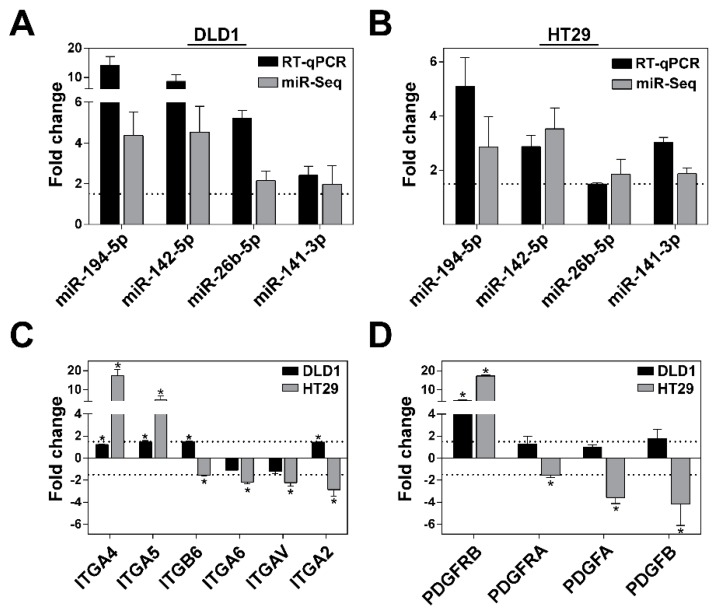
Validation of microenvironment-dependent gene and miRNA expression patterns colorectal carcinoma DLD1 and HT29 cells grown in MCS. (**A,B**) Validation of miRNA deep sequencing data. Graph showing the microenvironment-dependent miRNA expression in DLD1 (A) and HT29 (**B**) cells cultivated in MCS compared to expression levels in cells cultivated in 2D. RT-qPCR data were normalized using U6 snRNA gene as an internal control. (**C**) Graph representing the expression of genes encoding integrin subunits and (**D**) platelet-derived growth factors and its receptors in DLD1 and HT29 cells cultivated in MCS compared to expression levels in cells cultivated in 2D. TBP gene was used as the housekeeping gene for data normalization. * *p* < 0.05. 2D—monolayer cell culture; MCS —multicellular spheroid culture; miR-seq—data obtained by deep sequencing; RT-qPCR—data obtained by quantitative PCR.

To confirm the results of miRNA NGS data analysis, RT-qPCR was applied to assess the expression changes of 4 miRNAs including miR-194-5p, miR-142-5p, miR-26b-5p and miR-141-3p in CRC cells grown in MCS compared to the expression levels observed in cells cultivated in 2D. Although the magnitude of the expression changes slightly varied between two methods, the overall direction of miRNA expression changes observed by RT-qPCR data was consistent with the miRNA expression results obtained by NGS (Figure 4A,B).

To investigate possible relations between the miRNAs differentially expressed in CRC cells in an environment-dependent manner and their target genes, we evaluated the expression changes of genes associated with cell–ECM interactions and gap junction categories including integrins ITGA2, ITGA4, ITGA5, ITGA6, ITGA8, ITGAV, ITGB6, and ITGB8 and platelet-derived growth factors PDGFA, PDGFB, PDGFC as well as its receptors PDGFRA and PDGFRB in CRC cells grown in MCS vs 2D. RT-qPCR data analysis indicated that HT29 cells cultivated in MCS exhibited considerably more statistically significant (fold change >1.5, *p*-value < 0.05) changes of gene expression than DLD1 cells (Figure 4C,D). The expression of ITGA4 and ITGA5 was significantly up-regulated in HT29 cells, whereas the expression of ITGB6, ITGA6, ITGA2, ITGAV was down-regulated. Meanwhile, the expression of only ITGA5 and ITGB6 genes was significantly up-regulated in DLD1 cells. The expression analysis of platelet-derived growth factors and its receptors demonstrated a significant up-regulation of beta-type platelet-derived growth factor receptor PDGFRB in both cell lines grown in MCS. In addition, a significant down-regulation of PDGFA, PDGFB, and PDGFRA gene expression was observed in HT29 cells cultivated in MCS (Figure 4D).

### 3.6. miR-142-5p is a Diagnostic Biomarker of Rectal Cancer Following Neoadjuvant Long-Course Treatment

To investigate the potential diagnostic utility of miRNAs differentially deregulated in cells grown in MCS, we selected five miRNAs, miR-26b-5p, miR-142-5p, miR-194-5p, miR-301a-5p and miR-3074-5p, which were differentially expressed in both cell lines grown in MCS (Figure 1D). RT-qPCR was applied to assess the expression levels of selected miRNAs in tumor samples (*n* = 72) collected from 24 patients. The sample cohort contained three groups and included rectal tumor samples collected from patients before long course neoadjuvant treatment (*n* = 24), and normal (*n* = 24) and tumor tissue (*n* = 24) samples collected from the same patients after the therapy.

**Figure 5:**
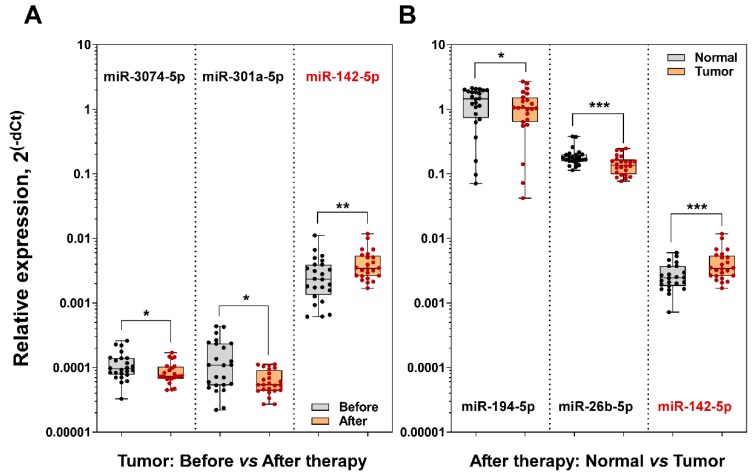
Evaluation of miR-26b-5p, miR-142-5p, miR-194-5p, miR-301a-5p and miR-3074-5p expression levels in rectal tumor tissue samples collected from patients who received long-course neoadjuvant treatment. The sample cohort was collected from 24 patients diagnosed with rectal cancer and contained three groups and included rectal tumor samples collected from patients before long course neoadjuvant treatment (*n* = 24), and normal (*n* = 24) and tumor tissue (*n* = 24) samples collected from the same patients after the therapy. **(A)** Box plots representing the expression of miR-3074-5p, miR-301a-5p and miR-142-5p in rectal tumor tissues collected before and after the therapy. **(B)** Box plots representing the expression of miR-194-5p, miR-26b-5p and miR-142-5p in rectal tumor and adjacent normal tissue samples collected from the same patients after the therapy. RNU48 was used as the internal control for the data normalization. * *p* < 0.05; ** *p* < 0.01; *** *p* < 0.001.

First, we evaluated miRNA expression levels in tumor tissue samples collected before and after chemoradiotherapy treatment. Our analysis indicated that the expression of 3 miRNAs was deregulated in tumor tissues following treatment (Figure 5A). Noteworthy, we determined that the expression of miR-142-5p was up-regulated in tumor tissue samples during the chemoradiation as well. Meantime, the expression of miR-3074-5p and miR-301a-5p was down-regulated in the treated tumor tissue sample group compared to tumor samples collected before the treatment. There was no difference in expression of miR-26b-5p and miR-194-5p in tumor tissue samples between two conditions (data is not shown). Next, we compared the expression levels of selected miRNAs in samples collected after chemoradiotherapy: tumor versus normal tissue samples. The analysis revealed that 3 miRNAs were differentially expressed in the rectal tumor tissue group (Figure 5B). We found that miR-194-5p and miR-26b-5p were significantly down-regulated, meanwhile, the expression of miR-142-5p was significantly upregulated in tumor tissue samples compared to normal rectum tissue. There was no difference in expression of miR-301a-5p and miR-3074-5p between normal and tumor tissue samples (data is not shown).

## 4. Discussion

A recent discovery of specific cancer–related dysregulation of miRNAs remains a promising area in the development of new reliable methods for cancer detection and prognosis [6]. However, commonly evaluated miRNAs as biomarkers remain broadly nonspecific and variable, weakly reproducible and which sensitivity is limited by various methodological challenges [29]. In addition, patient samples reduce the availability of biomaterial for large-scale –omics-based biomarker discovery. Our previous findings suggested that 3D cell culture should be considered as a critical experimental approach to uncover the regulation of genes and miRNA involved in tumor microenvironment-driven molecular processes in vivo [11]. We showed that CRC cells grown in laminin rich extracellular matrix or multicellular spheroid culture demonstrated distinct gene expression patterns compared to cells grown in 2D, including the up-regulated expression of cancer stem cell and endothelial-mesenchymal transition markers [30,31]. Furthermore, we revealed that radiation treatment resulted in different genome-wide gene expression profiles DLD1 and HT29 cells grown under 3D culture conditions compared to 2D [32]. Therefore, the employment of in vitro models that can restore characteristic in vivo conditions might evade these restrictions. MiRNAs, which are deregulated in tumor cells during cancer development, are proposed as useful biomarkers for cancer diagnosis and prognosis. Taking this into account, in this study we suggest a hypothesis that a better insight on the miRNA expression patterns in colorectal carcinoma cells grown in 3D cell culture could be applied for further evaluation of new biomarkers.

In our current study, the high throughput small RNA sequencing data analysis has elucidated that expression of the majority of miRNAs was exclusively deregulated in DLD1 cells. Changes in cancer cell surrounding microenvironment significantly affected the expression of 139 miRNAs in DLD1 cells and 32 miRNAs in HT29 cells grown under 3D cell culture conditions compared to 2D. Globally, this could be attributed to the distinct cellular origin, mutational status and characteristic tumorigenic properties of particular cell lines [33,34,35], which could lead to 3D cell culture-specific miRNA expression patterns. Besides, as depicted in Figure 1A, the changes in cellular morphology were more eminent to DLD1 cells during cellular adaptation to MCS. Although reports concerning the association between miRNA expression levels and cellular morphology are scarce, this observation is supported by the previous study indicating a clear correlation of miR-200c expression levels and morphology of gastric cancer cell lines [36], suggesting that higher differential expression of miRNAs could be associated with greater molecular rearrangements during microenvironment transition as well. In addition, we have previously shown that a higher number of genes were differentially expressed in HT29 cells grown in laminin-enriched 3D cell culture compared to DLD1 [31]. Our present analysis indicated higher alterations of target gene expression in HT29 cells as well suggesting gentle correlations between a number of genes and miRNAs differentially expressed in CRC cells grown in MCS. Furthermore, among these, the expression of 75% miRNAs was up-regulated in DLD1 and 66%—in HT29 cells, respectively, compared to miRNA expression levels in CRC cells grown in 2D. The previous study by Hwang et al. has demonstrated that a global up-regulation of miRNAs is initiated by the increased formation of cell–cell contacts in densely cultivated cells [37]. Due to this phenomenon, denoted as contact inhibition, gene translation processes are reduced resulting in cell cycle arrest and reduced cell proliferation [38]. Furthermore, following the growth in MCS, both cell lines exhibited a subset of 16 differentially expressed miRNAs. The significance of our observations is supported by previous reports indicating that miR-200a-3p [39], miR-141-3p [40], miR-375 [41], miR-194-5p [42], miR-27a-3p [43] and miR-29b-3p [42] could be clinically applied as potential CRC biomarkers. In addition, miR-194 was shown as one of the most deregulated miRNAs in cancerous tissue as well as miRNAs of the miR-200 family, including miR-200b and miR-200c [44]. Furthermore, the up-regulation of miR-10b-5p expression characterizes the tumors located in colon and rectum [45] which was coincided with the aberrant expression of this miRNA in CRC cells cultivated in multicellular spheroids. Therefore, these results show that the miRNA expression profiles of CRC cells cultivated in 3D spheroid culture could represent the miRNA expression levels in vivo and subsequently could be applied for wider clinical analysis to determine further clinical applications as potential biomarkers for CRC.

Previous studies highlighted that multicellular spheroids could restore natural cell-matrix interactions via a synthesis of their own ECM [46]. ECM could act not only as a major structural component for cancer cells but also could work as a signaling platform that allows selective signal transduction via cell–ECM interactions during the further formation of MCS [47]. Accordingly, our KEGG enrichment analysis demonstrated that cell adhesion-related processes including regulation of ECM-receptor interactions were most significantly enriched in miRNA target genes. For instance, we observed significant interactions between miR-29a/b/c and collagens (COL1A1, COL2A1, COL4A1/2, COL5A1/2/3), laminins (LAMA2, LAMC2) and integrins (ITGA6, ITGB1). Recent studies have shown a critical involvement of miR-29, which was demonstrated as a prognostic marker in colon cancer, in the regulation of ECM related gene expression homeostasis [48,49]. On the other hand, tumor cells cultivated in a 3D environment exhibit differential expression of integrins due to diminished cell contact with the surface [50,51]. Altered integrin expression in the colorectal cancer tissues promotes cell proliferation and invasiveness and a significant over-expression of integrins is associated with poor prognosis [52]. These results suggest that the activation of integrin signaling cascades may also regulate key molecules involved in the response to ECM microenvironment by regulating the expression of the corresponding miRNAs [53]. In addition, our KEGG analysis revealed that cell adhesion processes were enriched in putative miRNA target genes encoding platelet-derived growth factors (PDGFs) as well as its receptors (PDGFRs). Regarding the roles of genes encoding PDGF and its receptors in CRC development, it has been elucidated that CRC tissue exhibits up-regulated expression of PDGFRB which results in increased cell proliferation and epithelial-mesenchymal transition [54]. High levels of PDGFA and PDGFB in CRC tissues were shown to correlate with poor cancer prognosis as well [55]. Furthermore, the results of in silico target-gene analysis were further confirmed by qPCR analysis that indicated a differential expression of selected target genes in CRC cells cultivated in multicellular spheroids compared to gene expression levels in cells grown in 2D. Altogether these findings indicate that key miRNAs expressed in a microenvironment-dependent manner are involved in regulation of essential genes responsible for direct cell-microenvironment interactions and signal transduction in CRC cell lines.

Furthermore, we found that miRNAs differentially expressed in CRC cells grown in 3D were located in overall 19 polycistronic clusters indicating that the regulation of many microenvironment related miRNAs could be functionally coordinated in a physically adjacent manner. Of these, the miR-23a ∼27a ∼24-2 cluster, which encodes a pri-miRNA transcript consisting of three miRNAs—miR-23a, miR-27a, and miR-24-2, was differentially expressed in both CRC cell lines cultivated in 3D multicellular spheroids compared to 2D. However, the biological behavior of miRNAs clustered in miR-23a/27a/24-2 remains contradictory in tumor development and depends on specific tissue. According to previous reports, clustered miRNAs act as onco-miRs in breast cancer and stimulates the progression of cancer development [56], whereas the tumor-suppressive role of the miR-23a/27a/24-2 cluster has been reported in acute leukemia [57]. Meanwhile, in CRC, the levels of miR-23a and miR-27a are up-regulated in primary colorectal cancer tumors, as well as in human colorectal cancer cell lines and stem-like cancer cells [58]. Furthermore, Jin et al. demonstrated that miR-23a/27a/24-2 is the most up-regulated miRNA cluster in CRC under hypoxia conditions and is a critical regulator during a metabolic switch from oxidative phosphorylation to glycolysis [59]. Regarding these observations, our miRNA target analysis revealed key target gene groups implicated in cell adhesion including focal adhesion and proteoglycans in cancer associated with all miR-23a/27a/24-2 cluster miRNAs indicating that pro-oncogenic functions of these miRNAs including the promotion of tumor invasiveness and proliferation could be consolidated by the tumor microenvironment.

In this study, we demonstrated for the first time that miR-142-5p expression levels are significantly increased in rectum tumor tissue samples following long-course neoadjuvant treatment. miR-142-5p, a putative prognostic marker of poor colorectal cancer prognosis [60], has been shown overexpressed in colorectal tumor tissues in some studies [61,62,63]. Moreover, the overexpression of miR-142-5p was observed in colorectal cancer patients who received chemotherapy compared to expression levels in the group without the treatment [64], suggesting that the evaluation of miR-142-5p expression levels could serve as a diagnostic biomarker of cancer therapy. Next, our study revealed the down-regulation of miR-3074-5p and miR-301a-5p in rectal cancer tumor tissues after the therapy as well. Previously, the deregulated expression of these miRNAs was documented in colorectal cancer patient serum using high-throughput small RNA sequencing [65,66]. However, the diagnostic value of miR-3074-5p and miR-301a-5p remains unclear. Furthermore, we showed that the up-regulation of miR-142-5p remained in tumor tissue compared to adjacent normal rectum tissue samples collected from the same patients after the treatment. Besides, levels of miR-194-5p and miR-26b-5p were significantly lower in rectal cancer tissue compared to the normal rectum as well. Our results were consistent with previous reports showing down-regulation of miR-194-5p and miR-26b-5p in colorectal cancer tissues [14,67]. Altogether these results consolidate our findings in vitro. Chemotherapy and radiotherapy remain as a core of anticancer therapy, therefore, further development and validation of predictive assays using such in vitro 3D cell culture tumor models are highly needed.

## 5. Conclusions

Our findings suggest that miRNA expression patterns in CRC cells grown in 3D cell culture conditions could potentially resemble clinical significance of 3D cell culture model and a link between deregulated expression of miRNAs in CRC cells grown in 3D and miRNAs implicated in tumor development *in vivo*. These results indicate that 3D microenvironment-dependent deregulation of miRNA expression in CRC cells observed in our study potentially triggers essential molecular mechanisms predominantly including the regulation of cell adhesion, cell–cell and cell–ECM interactions important in CRC initiation and development. Furthermore, we demonstrated that miR-142-5p could serve as a diagnostic biomarker of cancer therapy. Therefore, our present results raise a necessity for a further clinical examination to evaluate the potential value of employed experimental design and detected miRNA signature for future biomarker discovery.

## Figures and Tables

**Table 1:** Patient demographic and clinical characteristics.

Factor		Total	%
**Age (median, range)**	68 (50–90)		
Sex	Male Female	15 9	62,5 37,5
Stage	3 4	23 1	95,8 4,2
T stage	Unknown T1 T3 T3/4 T4	1 1 15 1 6	4,2 4,2 62,5 4,2 25
N stage	Unknown N0 N1 N2	1 1 11 11	4,2 4,2 45,8 45,8
M stage	Unknown M0	3 21	12,5 87,5

**Table 2:** Clustered miRNAs differentially expressed in DLD1 and HT29 cells cultivated in multicellular spheroids.

Cell line	Chromosome	miRNA Cluster	Regulation
**DLD1**	1	miR-200a/b/429	**↑**
		miR-30c-1/30e	**↑**
		miR-181-a1/b1	**↑**
		miR-29c/29b-2	**↑**
	3	miR-425/191	**↑**
	7	miR-182/96/183	**↑**
		miR-29a/29b-1	**↑**
	8	miR-30b/30d	**↑**
	9	let-7a/let-7f-1/let-7d	**↑**
		miR-23b/27b/24-1	**↑**
		miR-181a-2/181b-2	**↑**
	11	miR-192/194-2/6750/6749	**↑**
	12	miR-200c/141	**↑**
	13	miR-17/18a/19a/20a/19b-1/92a-1	**↑**
	19	miR-24-2/27a/23a	**↑**
	22	let-7a/4763/let-7b	**↑**
	X	miR-221/222	**↓**
		miR-532/188/500a/362/501/500b/660/502	**↓**
**HT29**	1	miR-215/194	**↑**
	19	miR-23a/27a/24-2	**↑**

Note: miRNA cluster was denoted as differentially expressed if at least two clustered leading strand miRNAs were significantly up- or down-regulated in a 3D-dependent manner.

## Supplementary Materials

The following are available online at https://www.mdpi.com/2218-273X/10/4/613/s1, Additional file 1: Table S1: Primer sequences for miRNA cDNA synthesis, Table S2: Primer sequences for miRNA sequencing validation with RT-qPCR, Table S3: Primer sequences used for RT-qPCR; Additional file 2: Figure S1. miR-Seq read data quality control, Table S1: High-throughput miRNA sequencing read count summary, Table S2: Top 10 up-regulated and down-regulated miRNAs in DLD1 and HT29 cells cultivated in 3D multicellular spheroids compared to miRNA levels in cells grown in 2D: Additional file 3: Differential miRNA expression analysis of DLD1 and HT29 cell samples; Additional file 4: Table S1: KEGG signaling pathways enriched in experimentally validated and predicted target genes of differentially up-regulated miRNAs in DLD1 and HT29 cells grown in 3D, Table S2: Putative miRNA target genes associated with regulation of ECM-receptor interaction and formation of gap junctions; Additional file 5: Table S1: KEGG signaling pathways enriched in experimentally validated and predicted target genes of differentially expressed miRNAs in both DLD1 and HT29 CRC cell lines cultivated in multicellular spheroids compared to 2D, Table S2: Experimentally validated and predicted target genes of differentially expressed miRNAs in DLD1 and HT29 cells, which are implicated in molecular mechanisms regulating cell adhesion and CRC development; Additional file 6: Table S1: A list of miRNA clusters differentially expressed in DLD1 and HT29 cells cultivated in multicellular spheroids compared to miRNA levels in cells grown in 2D; Table S2: The expression pattern of miRNAs located in miR-23a/27a/24-2 polycistronic cluster in CRC cells grown in MCS; Table S3: KEGG categories enriched in experimentally validated and predicted target genes of miRNAs located in miR-23a/27a/24-2 cluster.
