# Supplementary material for: 3D Cell Culture-Based Global miRNA Expression Analysis Reveals miR-142-5p as a Theranostic Biomarker of Rectal Cancer Following Neoadjuvant Long-Course Treatment
Source: Biomolecules. 2020 Apr 16;10(4):613. doi: 10.3390/biom10040613 (PMC7226077; doi:10.3390/biom10040613)
Supplement: Supplementary file 1 [file biomolecules-10-00613-s001.zip › Supplementary Materials/biomolecules-756630supp1.pdf]

Additional File 1

Table S1. Primer sequences for miRNA cDNA synthesis.

miRNA	Stem-loop sequence
miR-194-5p	5'-GTCGTATCCAGTGCAGGGTCCGAGGTATTCGCACTGGATACGAC TCCACA-3'
miR-142-5p	5'-GTCGTATCCAGTGCAGGGTCCGAGGTATTCGCACTGGATACGAC AGTAGT-3'
miR-26b-5p	5'-GTCGTATCCAGTGCAGGGTCCGAGGTATTCGCACTGGATACGAC ACCTAT-3'
miR-141-3p	5'-GTCGTATCCAGTGCAGGGTCCGAGGTATTCGCACTGGATACGAC CCATCT-3'
miR-301a-5p	5'-GTCGTATCCAGTGCAGGGTCCGAGGTATTCGCACTGGATACGACAGTAGT-3'
miR-3074-5p	5'-GTCGTATCCAGTGCAGGGTCCGAGGTATTCGCACTGGATACGACCTGGCT-3'
U6	5'-GTCGTATCCAGTGCAGGGTCCGAGGTATTCGCACTGGATACGACAAAAATATG-3'
RNU48	5'- GTCGTATCCAGTGCAGGGTCCGAGGTATTCGCACTGGATACGACGGTCAGAGC-3'

Table S2. Primer sequences for miRNA sequencing validation with RT-qPCR.

miRNA	Forward sequence	Reverse sequence
miR-194-5p	5'-GGCCTGTAACAGCAACTCCA-3'	5'-CAGTGCAGGGTCCGAGGT-3'
miR-142-5p	5'-CGGCGGCATAAAGTAGAAAGC-3'	5'-CAGTGCAGGGTCCGAGGT-3'
miR-26b-5p	5'-CGGCGGTTCAAGTAATTCAGG-3'	5'-CAGTGCAGGGTCCGAGGT-3'
miR-141-3p	5'-CGGCGTAACACTGTCTGGTAA-3'	5'-CAGTGCAGGGTCCGAGGT-3'
miR-301a-5p	5'-GGCCGCTCTGACTTTATTGC-3'	5'-CAGTGCAGGGTCCGAGGT-3'
miR-3074-5p	5'-CGGTTCCTGCTGAACTG-3'	5'-CAGTGCAGGGTCCGAGGT-3'
U6	5'-GCGCGTCGTGAAGCGTTC-3'	5'-CAGTGCAGGGTCCGAGGT-3'
RNU48	5'-GCGTGCCATCACCGCAGC-3'	5'-CAGTGCAGGGTCCGAGGT-3'

Table 3. Primer sequences used for RT-qPCR.

Gene	Forward sequence	Reverse sequence
ITGA2	5\`-CCTACAATGTTGGTCTCCCAGA-3\`	5\`-AGTAACCAGTTGCCTTTTGGATT-3\`
ITGA4	5\`-AGCCCTAATGGAGAACCTTGT-3\`	5\`-CCAGTGGGGAGCTTATTTTCAT-3\`
ITGA5	5\`-GGCTTCAACTTAGACGCGGAG-3\`	5\`-TGGCTGGTATTAGCCTTGGGT-3\`
ITGA6	5\`-GGCGGTGTTATGTCCTGAGTC-3\`	5\`-AATCGCCCATCACAAAAGCTC-3\`
ITGA8	5\`-TCAGGCGTTCAACCTGGAC-3\`	5\`-GCGTCGGGTATGTGGAAGTC-3\`
ITGA9	5\`-TCGTTCTTCGGCTACGCAG-3\`	5\`-CCCCATCCACTCATCATCGC-3\`
ITGAV	5\`-ATCTGTGAGGTCGAAACAGGA-3\`	5\`-TGGAGCATACTCAACAGTCTTTG-3\`
ITGB6	5\`-CTCAACACAATAAAGGAGCTGGG-3\`	5\`-AAAGGGGATACAGGTTTTTCCAC-3\`
ITGB8	5\`-ACCAGGAGAAGTGTCTATCCAG-3\`	5\`-CCAAGACGAAAGTCACGGGA-3\`
PDGFA	5\`-GCAAGACCAGGACGGTCATTT-3\`	5\`-GGCACTTGACACTGCTCGT-3\`
PDGFB	5\`-CTCGATCCGCTCCTTTGATGA-3\`	5\`-CGTTGGTGCGGTCTATGAG-3\`
PDGFC	5\`-ATTCACAGCCCAAGGTTTCCT-3\`	5\`-GGGTCTTCAAGCCCAAATCTT-3\`
PDGFRA	5\`-TTGAAGGCAGGCACATTTACA-3\`	5\`-GCGACAAGGTATAATGGCAGAAT-3\`
PDGFRB	5\`-AGCACCTTCGTTCTGACCTG-3\`	5\`-TATTCTCCCGTGTCTAGCCCA-3\`
TBP	5\`-CCACTCACAGACTCTCACAAC-3\`	5\`-CTGCGGTACAATCCCAGAACT-3\`
