# Supplementary material for: 3D Cell Culture-Based Global miRNA Expression Analysis Reveals miR-142-5p as a Theranostic Biomarker of Rectal Cancer Following Neoadjuvant Long-Course Treatment
Source: Biomolecules. 2020 Apr 16;10(4):613. doi: 10.3390/biom10040613 (PMC7226077; doi:10.3390/biom10040613)
Supplement: Supplementary file 1 [file biomolecules-10-00613-s001.zip › Supplementary Materials/biomolecules-756630supp2.pdf]

# Additional File 2

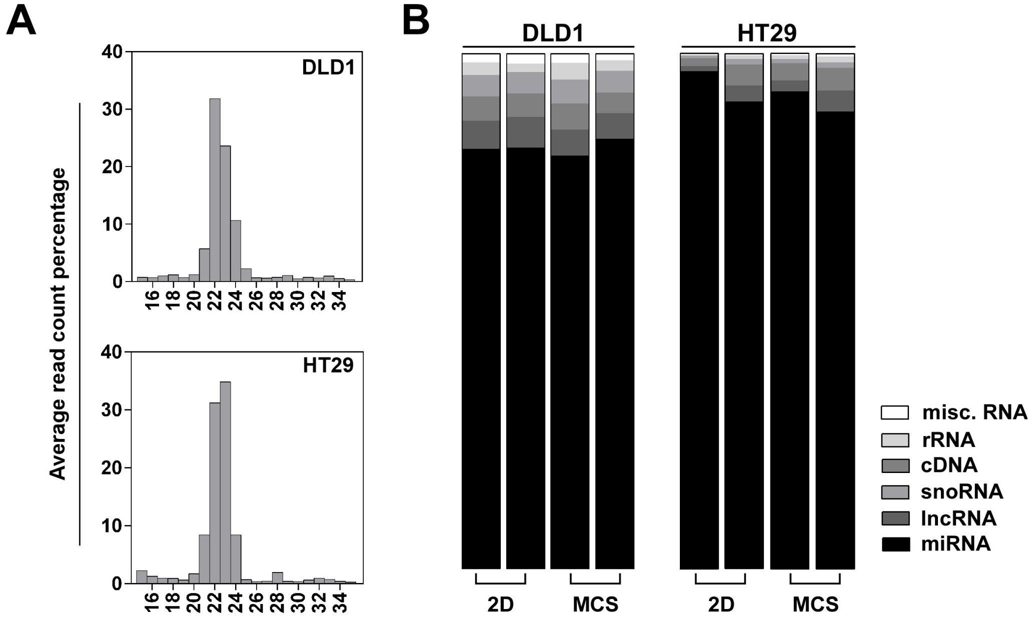


**Figure S1. miR-Seq read data quality control.** (**A**) Proportional distribution of read lengths in small RNA libraries, the total amount of sequenced reads was set to 100%. (**B**) Distribution of mapped reads, considering the total number reads mapped to genome as 100%.

**Table S1.** High-throughput miRNA sequencing read count summary.

| | Total read count | Total reads after QC | Reads mapped to human genome | Reads mapped to human miRNA |
|---|---|---|---|---|
| **DLD1_2D1** | 23 498 569 | 21 593039 (92%) | 21 252 619 (98%) | 15 814 475 (74%) |
| **DLD1_2D2** | 36 069 614 | 34 124874 (95%) | 33 579 814 (98%) | 25 477 315 (76%) |
| **DLD1_SF1** | 29 624 600 | 26 570728 (90%) | 26 171 877 (98%) | 18 069 336 (69%) |
| **DLD1_SF2** | 29 228 303 | 28 150644 (96%) | 27 745 559 (99%) | 20 463 716 (74%) |
| **HT29_2D1** | 11 544 757 | 11 359998 (98%) | 11 185 452 (98%) | 10 495 634 (94%) |
| **HT29_2D2** | 33 207 481 | 32 377468 (98%) | 30 934 141 (96%) | 26 211 524 (85%) |
| **HT29_SF1** | 12 732 347 | 12 353231 (97%) | 12 026 782 (97%) | 10 745 232 (89%) |
| **HT29_SF2** | 23 718 678 | 22 652552 (96%) | 21 584 426 (95%) | 17 182 624 (80%) |

*Note:* sample name abbreviations correspond to cell line used for experiment (DLD1 or HT29) and cell culture of either monolayer (2D) or multicellular spheroids (SF). Last numeral in sample name denominates the number of biological replicate.

**Table S2.** Top 10 up-regulated and down-regulated miRNAs in DLD1 and HT29 cells cultivated in 3D multicellular spheroids compared to miRNA levels in cells grown in 2D.

| DLD1 | | | HT29 | | |
|---|---|---|---|---|---|
| miRNA | Fold Change | p value | miRNA | Fold Change | p value |
| **miR-1246** | 10.82 | 8.92e-14 | **miR-210-3p*** | 6.27 | 1.40e-05 |
| **miR-9-3p** | 8.55 | 2.02e-08 | **miR-215-5p** | 4.73 | 1.95e-04 |
| **miR-9-5p** | 8.11 | 2.01e-13 | **miR-375*** | 3.53 | 2.37e-04 |
| **miR-95-3p** | 7.80 | 7.26e-15 | **miR-142-5p*** | 3.53 | 3.96e-10 |
| **miR-10b-5p** | 5.23 | 1.85e-26 | **miR-194-5p*** | 2.85 | 3.59e-02 |
| **miR-192-5p** | 5.07 | 4.14e-26 | **miR-378c*** | 2.11 | 1.65e-05 |
| **miR-142-5p*** | 4.54 | 2.87e-18 | **miR-455-3p** | 2.10 | 2.46e-02 |

| | | | | | |
|---|---|---|---|---|---|
| **miR-194-5p*** | 4.37 | 6.06e-25 | **miR-29b-3p*** | 2.02 | 1.39e-03 |
| **miR-210-3p*** | 4.32 | 1.06e-27 | **miR-27a-3p** | 1.87 | 1.53e-19 |
| **miR-146a-5p** | 4.32 | 1.79e-14 | **miR-141-3p*** | 1.87 | 7.82e-07 |
| | | | | | |
| **miR-371b-3p** | -7.43 | 2.74e-18 | **miR-934** | -3.24 | 1.33e-06 |
| **miR-30a-5p** | -5.67 | 5.11e-25 | **miR-205-5p** | -2.47 | 2.56e-02 |
| **miR-5100** | -4.22 | 1.50e-34 | **miR-3677-3p** | -2.12 | 9.75e-09 |
| **miR-372-3p** | -3.93 | 1.89e-10 | **miR-452-5p** | -2.07 | 5.48e-07 |
| **miR-3187-3p** | -3.40 | 8.91e-16 | **miR-7974** | -2.02 | 2.82e-03 |
| **miR-139-5p** | -3.34 | 2.89e-17 | **miR-301a-5p*** | -1.80 | 1.75e-05 |
| **miR-7-5p** | -3.11 | 2.22e-06 | **miR-874-3p** | -1.75 | 2.77e-02 |
| **miR-760** | -2.93 | 5.05e-14 | **miR-589-5p*** | -1.67 | 1.47e-10 |
| **miR-3529-3p** | -2.91 | 1.76e-06 | **miR-16-2-3p** | -1.49 | 1.60e-02 |
| **miR-500a-5p** | -2.90 | 7.97e-07 | **miR-31-3p** | -1.48 | 1.57e-02 |

***Note:*** * indicates that significant expression change was observed in both cell lines cultivated in multicellular spheroids.
