# Supplementary material for: 3D Cell Culture-Based Global miRNA Expression Analysis Reveals miR-142-5p as a Theranostic Biomarker of Rectal Cancer Following Neoadjuvant Long-Course Treatment
Source: Biomolecules. 2020 Apr 16;10(4):613. doi: 10.3390/biom10040613 (PMC7226077; doi:10.3390/biom10040613)
Supplement: Supplementary file 1 [file biomolecules-10-00613-s001.zip › Supplementary Materials/biomolecules-756630supp4.pdf]

# Additional File 4

**Table S1.** KEGG signaling pathways enriched in experimentally validated and predicted target genes of differentially up-regulated miRNAs in DLD1 and HT29 cells grown in 3D.

| KEGG pathway | Database | DLD1 | | | HT29 | | |
|---|---|---|---|---|---|---|---|
| | | miRNAs | Genes | p value | miRNA | Genes | p value |
| ECM-receptor interaction | miRTarBase | 20 | 19 | 6.71e-05 | 4 | 8 | 9.80e-03 |
| | microT-CDS | 78 | 64 | 3.44e-05 | 14 | 47 | 2.17e-33 |
| Gap junction | miRTarBase | 29 | 22 | 4.89e-06 | 7 | 12 | 7.56e-05 |
| | microT-CDS | 84 | 68 | 6.10e-04 | 18 | 42 | 7.51e-03 |

Note: a significant pathway enrichment was considered if corrected p value was <0.05 and at least 5 target genes were assigned for a functional group.

**Table S2.** Putative miRNA target genes associated with regulation of ECM-receptor interaction and formation of gap junctions.

| KEGG pathway | Gene | Database | DLD1 | HT29 |
|---|---|---|---|---|
| ECM-receptor interaction | COL1A1 | miRTarbase | **miR-29b-3p**, miR-29c-3p | **miR-29b-3p** |
| | | microT-CDS | let-7f-5p, miR-3184-5p, miR-423-5p, miR-29a-3p, **miR-29b-3p**, miR-29c-3p, let-7a-5p, let-7c-5p, let-7b-5p | **miR-29b-3p** |
| | COL4A1 | miRTarbase | miR-29a-3p, **miR-29b-3p**, miR-29c-3p | **miR-29b-3p** |
| | | microT-CDS | let-7b-3p, miR-429, miR-628-5p, let-7f-5p, let-7a-3p, miR-320d, miR-374b-5p, miR-152-3p, miR-29a-3p, **miR-29b-3p**, miR-200c-3p, miR-148a-3p, miR-374a-5p, miR-200b-3p, miR-29c-3p, let-7a-5p, let-7c-5p, let-7b-5p | **miR-29b-3p** |
| | COL4A2 | miRTarbase | **miR-210-3p**, miR-29a-3p, **miR-29b-3p**, miR-29c-3p | **miR-210-3p, miR-29b-3p** |
| | | microT-CDS | miR-29a-3p, **miR-29b-3p**, miR-29c-3p | **miR-29b-3p** |
| | ITGA6 | miRTarbase | miR-29a-3p, **miR-29b-3p**, miR-29c-3p | **miR-29b-3p** |
| | | microT-CDS | let-7b-3p, miR-582-5p, miR-30c-5p, miR-30d-5p, miR-340-5p, let-7a-3p, **miR-141-3p**, miR-32-5p, miR-30b-5p, miR-200a-3p, miR-9-5p | miR-30b-5p, **miR-141-3p**, miR-200a-3p |
| | ITGA9 | miRTarbase | **miR-194-5p** | **miR-194-5p** |
| | | microT-CDS | miR-30c-5p, miR-30d-5p, miR-340-5p, miR-152-3p, miR-148a-3p, **miR-30b-5p**, miR-194-3p | **miR-30b-5p** |
| | ITGB1 | miRTarbase | miR-183-5p, **miR-29b-3p**, miR-9-3p | **miR-29b-3p** |
| | | microT-CDS | miR-30c-5p, miR-30d-5p, miR-183-5p, **miR-30b-5p**, miR-203b-3p, miR-9-3p | **miR-30b-5p** |
| Gap junction | EGFR | miRTarbase | let-7a-5p, miR-146a-5p, **miR-27a-3p**, miR-574-3p | **miR-27a-3p** |
| | | microT-CDS | miR-429, miR-340-5p, **miR-27a-3p**, miR-126-5p, miR-200c-3p, miR-200b-3p, **miR-27b-3p** | **miR-27a-3p** |
| | GRB2 | miRTarbase | **miR-200a-3p, miR-27a-3p** | **miR-200a-3p, miR-27a-3p** |
| | | microT-CDS | **miR-27a-3p**, miR-182-5p, miR-126-5p, **miR-27b-3p** | **miR-27a-3p** |
| | KRAS | miRTarbase | let-7a-5p, miR-152-3p, **miR-181a-5p**, miR-200c-3p, **miR-27a-3p**, miR-340-5p | **miR-27a-3p** |
| | | microT-CDS | miR-30c-5p, miR-30d-5p, miR-340-5p, miR-320d, **miR-27a-3p**, miR-126-5p, **miR-30b-5p**, miR-27b-3p, **miR-142-5p** | **miR-30b-5p**, **miR-142-5p**, **miR-27a-3p** |
| | MAP2K1 | miRTarbase | **miR-181a-5p**, miR-181b-5p, miR-34a-5p | **miR-181b-5p** |
| | | microT-CDS | miR-34a-5p, **miR-181a-5p** | miR-552-3p |
| | MAPK1 | miRTarbase | **miR-181a-5p**, miR-28-5p, **miR-335-5p** | **miR-335-5p** |
| | | microT-CDS | miR-17-3p, miR-628-5p, miR-769-5p, miR-320d, miR-140-3p, miR-181a-2-3p, **miR-142-5p** | **miR-142-5p**, miR-24-3p |
| | PDGFA | miRTarbase | **miR-29b-3p** | **miR-29b-3p** |
| | | microT-CDS | miR-30c-5p, miR-30d-5p, miR-200a-5p, miR-29a-3p, **miR-29b-3p**, **miR-30b-5p**, **miR-375**, miR-29c-3p | **miR-375**, **miR-29b-3p**, miR-30b-5p |
| | PDGFB | miRTarbase | **miR-29b-3p** | **miR-29b-3p** |
| | | microT-CDS | miR-29a-3p, **miR-29b-3p**, miR-29c-3p | **miR-29b-3p** |
| | PDGFC | miRTarbase | **miR-29b-3p** | **miR-29b-3p** |
| | | microT-CDS | miR-30c-5p, miR-30d-5p, miR-29a-3p, **miR-29b-3p**, **miR-30b-5p**, miR-29c-3p, **miR-142-5p** | **miR-142-5p**, **miR-30b-5p**, **miR-29b-3p** |
| | PDGFRA | miRTarbase | let-7b-5p, miR-146b-5p, **miR-29b-3p**, miR-34a-5p | **miR-29b-3p** |
| | | microT-CDS | miR-340-5p, miR-34a-5p, miR-374b-5p, **miR-27a-3p**, **miR-182-5p**, **miR-141-3p**, miR-126-5p, **miR-200a-3p**, **miR-27b-3p**, miR-203b-5p, miR-146a-5p, **miR-142-5p**, miR-9-3p, miR-146b-5p | **miR-142-5p, miR-200a-3p, miR-141-3p, miR-27a-3p** |

| | | | |
|---|---|---|---|
| PDGFRB | miRTarbase | - | **miR-29b-3p, miR-30b-5p** |
| | microT-CDS | - | miR-24-3p |

Note: bold script indicates a significant expression change of particular miRNA observed in both CRC cell lines.
