# Supplementary material for: 3D Cell Culture-Based Global miRNA Expression Analysis Reveals miR-142-5p as a Theranostic Biomarker of Rectal Cancer Following Neoadjuvant Long-Course Treatment
Source: Biomolecules. 2020 Apr 16;10(4):613. doi: 10.3390/biom10040613 (PMC7226077; doi:10.3390/biom10040613)
Supplement: Supplementary file 1 [file biomolecules-10-00613-s001.zip › Supplementary Materials/biomolecules-756630supp5.pdf]

Additional File 5

Table S1. KEGG signaling pathways enriched in experimentally validated and predicted target genes of differentially expressed miRNAs in both DLD1 and HT29 CRC cell lines cultivated in multicellular spheroids compared to 2D.

	microT-CDS			miRTarBase		
**KEGG pathway**	**miRNAs**	**Genes**	**p value**	**miRNAs**	**Genes**	**p value**
ECM-receptor interaction	13	45	2.74e-33	4	8	1.40e-02
Focal adhesion	15	109	2.24e-07	11	41	1.50e-15
Proteoglycans in cancer	15	89	2.84e-07	11	35	2.07e-15
Axon guidance	14	65	3.28e-06	7	18	5.90e-05
Renal cell carcinoma	14	40	6.66e-06	9	19	1.63e-12
Thyroid hormone signaling pathway	14	58	3.43e-05	9	20	6.00e-09
Rap1 signaling pathway	15	99	8.41e-05	11	29	2.05e-10
ErbB signaling pathway	13	48	1.23e-04	8	18	1.96e-09
Regulation of actin cytoskeleton	14	101	1.58e-04	8	22	7.26e-06
PI3K-Akt signaling pathway	15	149	2.16e-04	11	51	1.50e-15
Ras signaling pathway	15	98	2.84e-04	11	28	6.00e-09
TGF-beta signaling pathway	13	40	3.89e-04	8	15	3.45e-07
Platelet activation	15	60	3.89e-04	8	17	1.69e-06
Amoebiasis	14	47	4.24e-04	5	12	3.00e-04
Glioma	15	32	4.59e-04	11	23	1.50e-15
Pathways in cancer	16	171	5.60e-04	12	62	1.50e-15
Hippo signaling pathway	15	59	1.09e-03	8	22	2.20e-08
Signaling pathways regulating pluripotency of stem cells	14	65	1.26e-03	10	25	2.24e-11
Neurotrophin signaling pathway	15	60	1.46e-03	10	19	5.51e-08
Wnt signaling pathway	14	64	1.57e-03	9	19	7.94e-07
Estrogen signaling pathway	14	43	1.57e-03	8	18	1.53e-08
Viral carcinogenesis	15	77	1.80e-03	10	24	2.19e-07
Colorectal cancer	14	32	4.09e-03	9	25	1.50e-15
FoxO signaling pathway	14	61	5.25e-03	12	30	1.50e-15
Prolactin signaling pathway	13	34	5.35e-03	9	19	6.59e-12
AMPK signaling pathway	14	58	1.62e-02	8	12	2.04e-03
mTOR signaling pathway	13	31	2.31e-02	9	15	4.17e-04
Gap junction	14	38	2.31e-02	6	11	3.91e-04
Prostate cancer	15	41	2.31e-02	11	32	1.50e-15
Melanoma	15	36	2.31e-02	11	23	1.50e-15
Non-small cell lung cancer	14	26	2.54e-02	10	16	1.05e-10
Pancreatic cancer	15	30	2.76e-02	11	24	1.50e-15
Acute myeloid leukemia	13	27	2.78e-02	8	12	1.09e-06
Choline metabolism in cancer	13	46	2.78e-02	10	20	3.58e-10
Oxytocin signaling pathway	15	65	2.78e-02	7	13	5.43e-03
T cell receptor signaling pathway	12	47	3.00e-02	7	16	1.11e-06
cAMP signaling pathway	14	82	3.14e-02	8	18	2.70e-04
Fc gamma R-mediated phagocytosis	13	42	3.17e-02	7	10	2.50e-03
Small cell lung cancer	14	40	3.23e-02	12	23	1.84e-14
Bacterial invasion of epithelial cells	12	36	3.29e-02	7	12	2.88e-05
cGMP-PKG signaling pathway	15	69	3.29e-02	7	12	2.24e-02
Sphingolipid signaling pathway	14	48	4.32e-02	10	15	2.88e-05

Note: significant enrichment was considered if corrected p value was <0.05 and at least 5 target genes were assigned for a functional group.

Table S2. Experimentally validated and predicted target genes of differentially expressed miRNAs in DLD1 and HT29 cells, which are implicated in molecular mechanisms regulating cell adhesion and CRC development.

			**Cell adhesion**				**Signaling pathways regulating CRC development**							
			**ECM-receptor interaction**		**Gap junction**		**PI3K-Akt signaling pathway**		**Ras signaling pathway**		**TGF-beta signaling pathway**		**Wnt signaling pathway**	
**Fold change**		**p value**	1.40e-02	2.74e-33	3.91e-04	2.31e-02	0.00e+00	2.16e-04	6.00e-09	2.84e-04	3.45e-07	3.89e-04	7.94e-07	1.57e-03
**DLD1**	**HT29**	**miRNA**	Validated	Predicted	Validated	Predicted	Validated	Predicted	Validated	Predicted	Validated	Predicted	Validated	Predicted
4.54	3.53	miR-142-5p	n.s.	3	n.s.	10	n.s.	28	n.s.	25	n.s.	11	n.s.	9
4.37	2.85	miR-194-5p	1	1	n.s.	2	4	10	2	5	2	3	2	8
4.32	6.27	miR-210-3p	1	n.s.	n.s.	n.s.	2	n.s.	1	n.s.	n.s.	n.s.	n.s.	n.s.
2.43	1.54	miR-200a-3p	n.s.	4	1	9	6	24	2	15	2	6	4	11
2.36	3.53	miR-375	n.s.	n.s.	n.s.	5	6	10	3	7	2	4	3	5
2.18	1.52	miR-30b-5p	n.s.	6	1	10	4	27	1	20	1	6	1	14
2.16	1.85	miR-26b-5p	n.s.	5	n.s.	4	5	22	2	15	n.s.	10	n.s.	13
2.02	2.02	miR-29b-3p	6	18	5	4	22	33	10	14	5	n.s.	3	5
1.97	1.87	miR-141-3p	n.s.	4	n.s.	10	5	24	3	15	2	7	3	11
1.89	1.87	miR-27a-3p	n.s.	16	3	12	8	48	6	31	2	12	2	19

1.80	1.51	miR-181b-5p	n.s.	1	2	5	6	12	3	6	n.s.	3	1	4
1.67	1.82	miR-335-5p	1	n.s.	1	1	5	1	3	4	5	n.s.	3	n.s.
1.59	1.46	miR-3074-5p	n.s.	3	n.s.	1	n.s.	12	n.s.	12	n.s.	6	n.s.	2
1.49	2.11	miR-378c	n.s.	1	n.s.	n.s.	n.s.	4	n.s.	4	n.s.	3	n.s.	3
-1.47	-1.67	miR-589-5p	n.s.	2	n.s.	2	n.s.	9	n.s.	6	n.s.	1	n.s.	6
-1.83	-1.80	miR-301a-5p	n.s.	1	n.s.	1	n.s.	5	n.s.	3	n.s.	1	n.s.	3

Note: significant enrichment was considered if corrected p value was <0.05 and at least 5 target genes were assigned for a functional group, n.s. – not significant.
