# Supplementary material for: 3D Cell Culture-Based Global miRNA Expression Analysis Reveals miR-142-5p as a Theranostic Biomarker of Rectal Cancer Following Neoadjuvant Long-Course Treatment
Source: Biomolecules. 2020 Apr 16;10(4):613. doi: 10.3390/biom10040613 (PMC7226077; doi:10.3390/biom10040613)
Supplement: Supplementary file 1 [file biomolecules-10-00613-s001.zip › Supplementary Materials/biomolecules-756630supp6.pdf]

Additional File 6

Table S1. A list of miRNA clusters differentially expressed in DLD1 and HT29 cells cultivated in multicellular spheroids compared to miRNA levels in cells grown in 2D.

Chromosome	Cluster	Clustred miRNA	Coordinates GRCh38	Fold Change	p value	Cell culture
1	miR-200a/b/429	miR-200b-3p	1167104-1167198[+]	2.39	1.02e-06	DLD1
		miR-200b-5p	1167104-1167198[+]	2.17	7.60e-06	DLD1
		miR-200a-3p	1167863-1167952[+]	2.43	2.78e-16	DLD1
		miR-200a-5p	1167863-1167952[+]	1.90	5.28e-05	DLD1
		miR-429	1169005-1169087[+]	1.48	4.87e-02	DLD1
	miR-30c-1/30e	miR-30e-3p	40754355-40754446[+]	1.58	6.31e-03	DLD1
		miR-30c-5p	40757284-40757372[+]	1.58	9.82e-09	DLD1
	miR-181-a1/b1	miR-181b-5p	198858873-198858982[-]	1.80	2.11e-13	DLD1
		miR-181b-3p	198858873-198858982[-]	1.49	4.49e-02	DLD1
		miR-181a-5p	198859044-198859153[-]	3.41	6.02e-26	DLD1
		miR-181a-3p	198859044-198859153[-]	1.72	5.28e-08	DLD1
	miR-29c/29b-2	miR-29c-3p	207801852-207801939[-]	2.53	2.85e-27	DLD1
		miR-29c-5p	207801852-207801939[-]	1.62	9.14e-03	DLD1
		miR-29b-3p	207802443-207802523[-]	2.02	9.56e-03	DLD1
3	miR-425/191	miR-425-3p	49020148-49020234[-]	1.51	4.53e-07	DLD1
		miR-191-5p	49020618-49020709[-]	1.70	2.17e-11	DLD1
7	miR-182/96/183	miR-182-5p	129770383-129770492[-]	1.96	1.36e-07	DLD1
		miR-183-5p	129774905-129775014[-]	1.66	6.36e-03	DLD1
	miR-29a/29b-1	miR-29a-3p	130876747-130876810[-]	1.99	2.11e-17	DLD1
		miR-29b-3p	130877459-130877539[-]	2.02	9.56e-03	DLD1
8	miR-30b/30d	miR-30b-5p	134800520-134800607[-]	2.18	2.93e-13	DLD1
		miR-30d-5p	134804876-134804945[-]	1.64	2.94e-03	DLD1
9	let-7a/let-7f-1/ let-7d	let-7a-5p	94175957-94176036[+]	2.98	2.00e-08	DLD1
		let-7a-3p	94175957-94176036[+]	1.79	4.13e-13	DLD1
		let-7f-5p	94176347-94176433[+]	1.62	7.32e-04	DLD1
	miR-23b/27b/24-1	miR-23b-3p	95085208-95085304[+]	3.88	2.02e-61	DLD1
		miR-27b-3p	95085445-95085541[+]	3.43	5.21e-24	DLD1
		miR-27b-5p	95085445-95085541[+]	2.86	1.25e-05	DLD1
		miR-24-3p	95086021-95086088 [+]	*-1.14*	*6.17e-01*	DLD1
	miR-181a-2/181b-2	miR-181a-5p	124692442-124692551[+]	3.41	6.02e-26	DLD1
		miR-181a-2-3p	124692442-124692551[+]	2.81	3.22e-31	DLD1
		miR-181b-5p	124693710-124693798[+]	1.80	2.11e-13	DLD1
11	miR-192/194-2/ 6750/6749	miR-192-5p	64891137-64891246[-]	5.07	4.14e-26	DLD1
		miR-194-5p	64891355-64891439[-]	4.37	6.06e-25	DLD1
		miR-194-3p	64891355-64891439[-]	4.11	1.31e-14	DLD1
		miR-6750	64898363-64898437 [-]	*-2.40*	*5.21e-01*	DLD1
		miR-6749	64902387-64902455 [-]	*2.96*	*3.13e-01*	DLD1
12	miR-200c/141	miR-200c-3p	6963699-6963766[+]	2.06	2.02e-05	DLD1
		miR-141-3p	6964097-6964191[+]	1.97	2.13e-02	DLD1
13	miR-17/18a/ 19a/ 20a/ 19b-1/92a-1	miR-17-3p	91350605-91350688[+]	1.49	4.60e-05	DLD1
		miR-18a-5p	91350751-91350821[+]	-1.53	4.32e-02	DLD1
		miR-19a-3p	91350891-91350972 [+]	*1.04*	*8.75e-01*	DLD1
		miR-20a	91351065-91351135 [+]	*1.35*	*1.77e-04*	DLD1
		miR-19b-3p	91351192-91351278 [+]	*1.53*	*9.22e-02*	DLD1
		miR-92a	91351314-91351391 [+]	*1.12*	*4.45e-01*	DLD1
19	miR-24-2/27a/23a	miR-27a-3p	13836440-13836517[-]	1.89	3.91e-07	DLD1
		miR-27a-5p	13836440-13836517[-]	-1.59	1.01e-02	DLD1
		miR-23a-3p	13836587-13836659[-]	1.48	6.41e-07	DLD1
		miR-24-3p	13836287-13836359 [-]	*-1.14*	*6.17e-01*	DLD1
22	let-7a/4763/let-7b	let-7a-5p	46112749-46112822[+]	2.98	2.00e-08	DLD1
		let-7a-3p	46112749-46112822[+]	1.79	4.13e-13	DLD1
		let-7b-5p	46113686-46113768[+]	4.28	7.05e-70	DLD1
		let-7b-3p	46113686-46113768[+]	1.47	2.27e-02	DLD1
		miR-4763	46113566-46113657 [+]	*-1.14*	*6.17e-01*	DLD1
X	miR-221/222	miR-221-5p	45746157-45746266[-]	-1.82	4.08e-03	DLD1
		miR-222-3p	45747015-45747124[-]	-1.93	1.22e-08	DLD1
	miR-532/188/ 500a/ 362/501/ 500b/ 660/502	miR-500a-3p	50008431-50008514[+]	-1.64	3.19e-05	DLD1
		miR-500a-5p	50008431-50008514[+]	-2.90	7.97e-07	DLD1
		miR-362-5p	50008964-50009028[+]	-1.69	1.00e-10	DLD1
		miR-501-3p	50009722-50009805[+]	-1.82	6.53e-04	DLD1
		miR-501-5p	50009722-50009805[+]	-2.27	9.93e-05	DLD1
		miR-532-5p	50003148-50003238 [+]	*-1.34*	*2.15e-04*	DLD1
		miR-188-5p	50003503-50003588 [+]	*-2.20*	*4.67e-02*	DLD1
		miR-500b-3p	50010672-50010750 [+]	*-17.27*	*2.70e-02*	DLD1
		miR-660-5p	50013241-50013337 [+]	*-1.39*	*3.85e-04*	DLD1
		miR-502-3p	50014598-50014683 [+]	*-1.47*	*5.16e-02*	DLD1
1	miR-215/194	miR-215-5p	220117853-220117962 [-]	4.73	1.95e-04	HT29
		miR-194-5p	220118157-220118241 [-]	2.85	3.59e-02	HT29

19	miR-24-2/27a/23a	miR-24-3p	13836287-13836359 [-]	1.71	8.41e-04	HT29
		miR-27a-3p	13836440-13836517 [-]	1.87	1.53e-19	HT29
		miR-23a-3p	13836587-13836659 [-]	*1.37*	*3.64e-01*	HT29

Table S2. The expression pattern of miRNAs located in miR-23a/27a/24-2 polycistronic cluster in CRC cells grown in MCS.

Cluster	Chromosome	miRNA	DLD1		HT29	
			Fold change	p value	Fold change	p value
miR-23a/27a/24-2	19	miR-23a-3p	**1.48**	**6.41e-07**	1.37	3.64e-01
		miR-24-3p	-1.14	6.17e-01	**1.71**	**8.41e-04**
		miR-27a-3p	**1.89**	**3.91e-07**	**1.87**	**1.53e-19**

Note: statistically significant miRNA expression changes detected in DLD1 and HT29 cells cultivated in multicellular spheroids compared to 2D are indicated in bold.

Table S3. KEGG categories enriched in experimentally validated and predicted target genes of miRNAs located in miR-23a/27a/24-2 cluster.

KEGG pathway	microT-CDS			miRTarBase		
	miRNAs	Genes	p value	miRNAs	Genes	p value
Proteoglycans in cancer	3	45	3.19e-04	3	13	1.79e-05
Rap1 signaling pathway	3	50	4.53e-03	3	9	7.63e-03
Hippo signaling pathway	3	28	2.59e-02	3	7	1.51e-02
Focal adhesion	3	47	2.69e-02	3	10	1.67e-03

Note: a significant enrichment was considered if corrected p value was <0.05 and at least 5 target genes were assigned for a functional group.
